# Transplantation of Photoreceptor and Total Neural Retina Preserves Cone Function in P23H Rhodopsin Transgenic Rat

**DOI:** 10.1371/journal.pone.0013469

**Published:** 2010-10-19

**Authors:** Ying Yang, Saddek Mohand-Said, Thierry Léveillard, Valérie Fontaine, Manuel Simonutti, José-Alain Sahel

**Affiliations:** 1 INSERM, U968, Paris, France; 2 Université Pierre et Marie Curie-Paris 6, UM80, Institut de la Vision, Paris, France; 3 CNRS, UMR_7210, Paris, France; 4 Centre Hospitalier National d'Ophtalmologie des Quinze-Vingts, INSERM-DHOS CIC 503, Paris, France; 5 Institute of Ophthalmology, University College of London, London, United Kingdom; 6 Fondation Ophtalmologique Adolphe de Rothschild, Paris, France; University of Washington, United States of America

## Abstract

**Background:**

Transplantation as a therapeutic strategy for inherited retinal degeneration has been historically viewed to restore vision as a method by replacing the lost retinal cells and attempting to reconstruct the neural circuitry with stem cells, progenitor cells and mature neural retinal cells.

**Methods and Findings:**

We present evidence for an alternative strategy aimed at preventing the secondary loss of cones, the most crucial photoreceptors for vision, by transplanting normal photoreceptors cells into the eye of the P23H rat, a model of dominant retinitis pigmentosa. We carried out transplantation of photoreceptors or total neural retina in 3-month-old P23H rats and evaluated the function and cell counts 6 months after surgery. In both groups, cone loss was significantly reduced (10%) in the transplanted eyes where the cone outer segments were found to be considerably longer. This morphological effect correlated with maintenance of the visual function of cones as scored by photopic ERG recording, but more precisely with an increase in the photopic b-wave amplitudes by 100% and 78% for photoreceptor transplantation and whole retinal transplantation respectively.

**Conclusions:**

We demonstrate here that the transplanted tissue prevents the loss of cone function, which is further translated into cone survival.

## Introduction

Retinitis pigmentosa (RP), the leading cause of inherited retinal blindness, encompasses a heterogeneous group of inherited disorders. RP is also called rod–cone dystrophy because of the sequential degeneration of rod and cone photoreceptors, though the causal mutations affect genes exclusively expressed in rods in the vast majority of cases. Following the loss of rod photoreceptors in animal models of RP, cone photoreceptors display abnormal morphology with shortening and disorganization of cone outer segments that correlate with functional deficit [1], [2].

A variety of therapeutical approaches has been attempted in RP. One classical method uses transplantation of retinal cells or retinal sheets to replace the missing photoreceptors and establish functional connections between the transplanted cells and the host inner retinal neurons [3]–[5]. The first attempt at transplantation using pieces of retinal tissue from salamander dates back to the early 1920s [6]. The first success was obtained in 1959 when fetal rat retinas were transplanted into the maternal eyes [7]. Since then, the surgical procedure has been initiated by performing subretinal transplantation of embryonic stem cells, progenitor cells, total retina, neural retinal cells, and photoreceptors. Different treatment modalities in animal models of retinal degeneration, including rodent fetal retina [8]–[12], human retinal pigment epithelium cells [13], [14] and human neural progenitor cells [15], [16] were extensively studied by Lund and coworkers during the last 40 years. In a series of studies, Aramant and Seiler showed that fetal retina can integrate into a degenerated retina, repair the damaged retinas [17], [18] and preserve visual function [19]–[24]. Indeed, they have observed new synapses between the grafted and host tissues, but the density of these connections should be higher and cover a larger surface in order to restore a more satisfactory visual function. In addition, formation of rosettes of photoreceptors from the transplant were often reported, from which the limited cell-cell integration between the grafted and host tissues could not contribute to an improved visual function, thus the visual restoration reported by these studies is likely to be related to a paracrine effect. This has been studied and demonstrated by Gamm et al. [15] by showing a survival paracrine effect after subretinal injection of wild type and genetically modified human neural progenitor cells in Royal College of Surgeons rats.

To become a clinical therapeutic strategy, the transplantation must fulfill the following criteria: 1) the transplant should survive in the host retina, 2) the outer segments of the transplanted photoreceptor cells should be in direct contact with the retinal pigment epithelium, 3) the photoreceptor cells of the transplant should be able to form synaptic connections with the host retina. These criteria seem difficult to achieve. While survival of the grafted tissue has been reported by many investigators, little evidence for integration of the grafts into the degenerated host retina, establishment of functional synaptic connections with other neurons in the host retina or restoration of the visual function has been provided so far. Survival of retinal progenitor grafts accompanied by preserved morphology and laminar organization of the host retina has been found by Chacko et al. [25], suggesting that progenitors may be a viable therapeutic option. To evaluate the migration and integration potential of the transplanted cells and their relation to their differentiation state, MacLaren et al., 2006 [26] studied transplantation of precursor cells at different stages of maturation defined by activation of the transcription factor Nrl (specific for post-mitotic rod precursors and persisting in adult rods). Surprisingly, they observed that successfully integrated rod photoreceptors are derived only from post-mitotic rod precursors (postnatal 1 to 7 days) but not from proliferating progenitor or stem cells, thus defining an optimal ontogenetic stage of donor cells for successful transplantation. In addition, the integrated cells were able to respond to light and make functional synaptic connections to downstream retinal targets. Though this study provides clear evidence that cell-replacement therapy may be possible in the future, the translation of its finding into an effective clinical practice will face the problem of availability of donor progenitor cells, as the period corresponding to the peak of rod genesis in humans is likely to be in the second trimester [27]. The use of human stem cell or induced pluripotent stem cells may represent an alternative, but a lot of works are still remaining in order to control and optimize the differentiation of these cells and to resolve the safety aspects, for example, the transcription factor regulation [28].

We proposed and established that retinal transplants might exert a paracrine neuroprotective effect on residual host cones. The present study was designed to extend our previous results obtained in *rd1* mouse (a relevant model of human recessive RP) to transgenic rhodopsin P23H mutant rat, a frequent mutation in dominant human disease and a good model for studying long-term effects and functional phenomena. The morphologic and functional assessment of the effect of either photoreceptor or total neural retinal transplantation in this study provide the first evidence for rescuing the cone photoreceptor function in an animal model of RP. This analysis also shed light on the possible mechanisms underlying the trophic interactions between photoreceptors. We found that the functional effect after transplantation was more significantly improved than the effect on cell counting, suggesting that cone survival may depend on the maintenance of cone function.

## Results

In transgenic P23H rat we previously showed that 41% of cones have been lost by the age of 3 to 6 months and a subsequent loss of 28% occurred between 6 and 9 months, while most of the rods have been found degenerated at 6 months of age [2]. In the same study, functional assessment performed by electroretinogram (ERG) recordings showed a loss of scotopic (rods) and photopic (cones) function matching photoreceptor degeneration. The average amplitude of the scotopic ERG b-wave was reduced by 89% between 2 and 9 months of age, while the photopic ERG b-wave amplitude was decreased by 77% during the same period [2]. This sequential pattern of degeneration of rods and cones makes the heterozygous P23H rat an appropriate model for evaluation of cone rescue strategies. In addition, the kinetics of photoreceptor degeneration in P23H rat allows long-term ERG recording.

**Figure 1:**
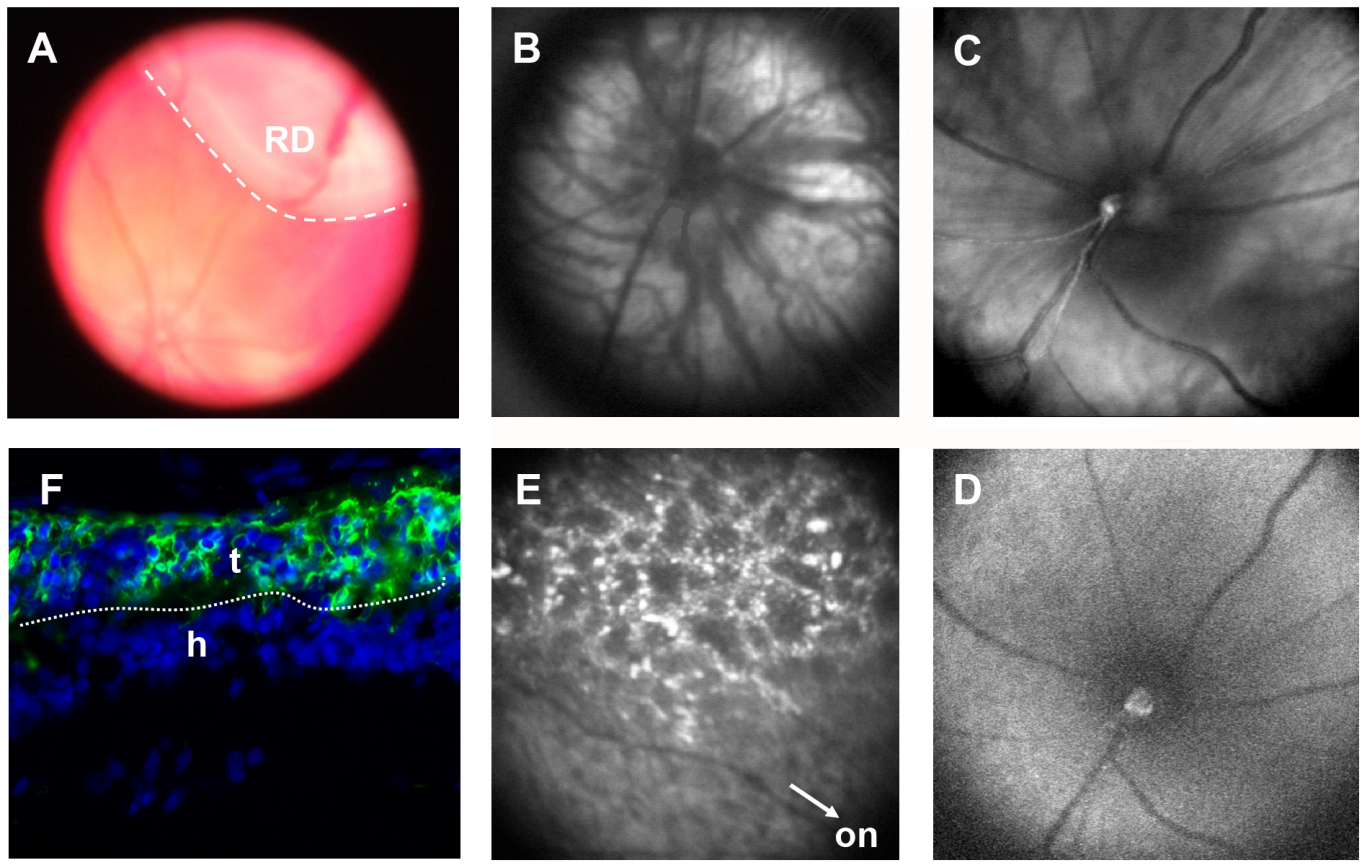
Visualization of the transplanted tissue. (**A**) A retinal detachment (RD, white line) was detectable immediately after transplantation from fundus of the transplanted eye in which the graft was placed just under the neural retina. (**B–E**) Photos of angiograph from P23H rat retinas. (**B** and **E**) Fundus photos from a transplanted eye after surgery. (**C** and **D**) Fundus photos from a control eye without transplantation. (**B** and **C**) Fundus photos taken with red-free light at 1 week after operation (Fundus of the transplanted retina was similar as that of the control, showing no detachment and hemorrhage). (**D** and **E**) Autofluorescence images taken at 2 months after operation. There was no hyper autofluorescence in the control retina (**D**), while it was observed in the transplanted retina (**E**), from transplant stained by PKH26 fluorescent cell linker in the superior of the optic nerve (ON, indicated by a white head arrow). (**F**) Vertical sections 6 months after transplantation showing the transplant (t) labeled with anti-rhodopsin antibody (green) apposed to the neural retina of the host (h).

**Figure 2:**
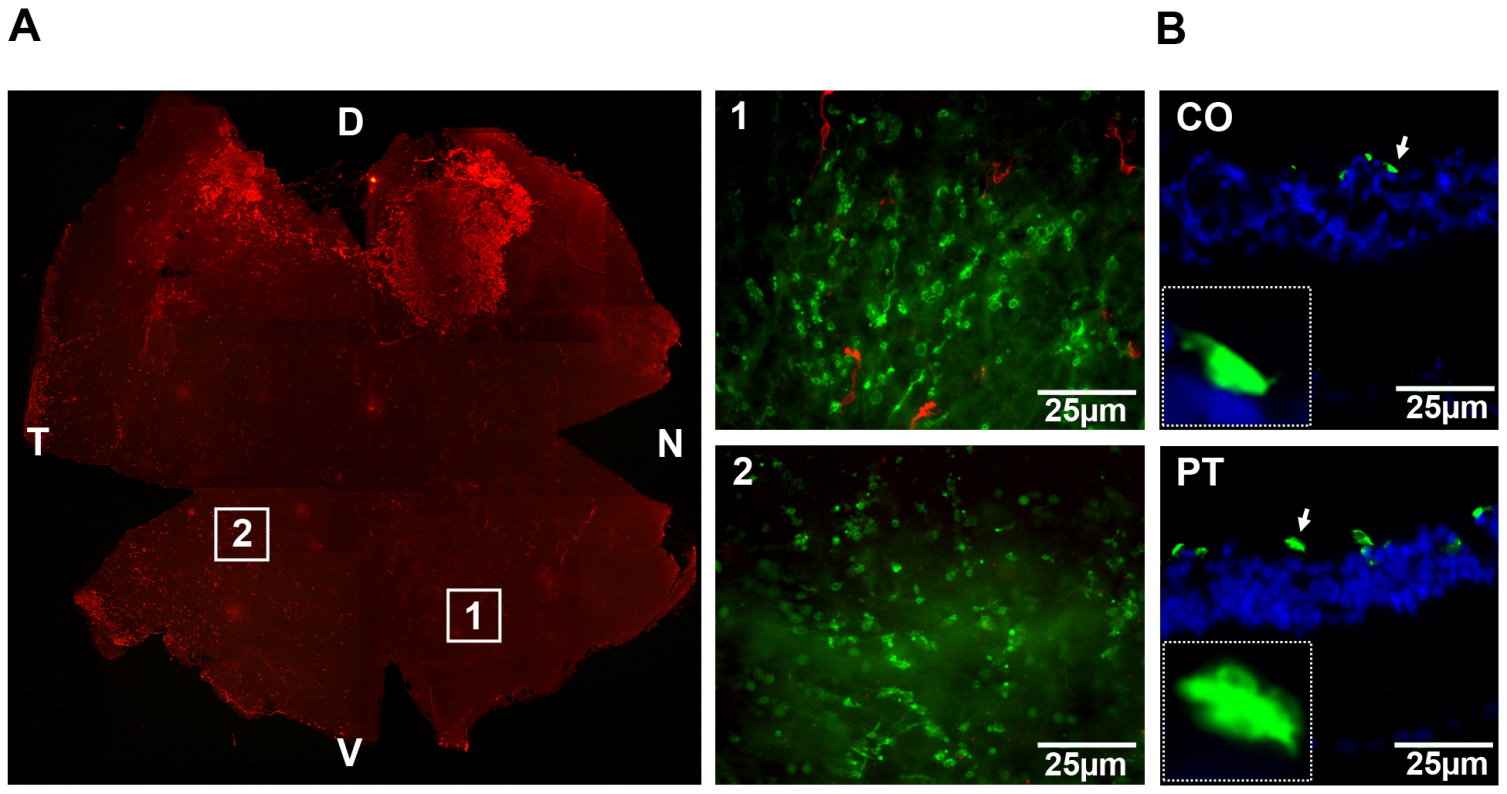
Effect of transplantation to P23H retinas at 9 months of age. (**A**) Immunolabeling of the flat-mounted degenerating retina (Rho-4D2 antibody, red) with retinal transplant. Due to degeneration, almost no rods were detected except the transplant in the dorsal host retina. Images1 and 2 were taken respectively in the nasal-ventral, temporal-ventral quadrants, both far from and opposite to the transplant. D: dorsal, V: ventral, N: nasal, T: temporal, PNA lectin: green, Rho-4D2 antibody: red. (**B**) Immunolabeling of cones with PNA lectin in the control and photoreceptor transplanted retinas from the same animal. The two images were taken in the same areas from the nasal-ventral quadrant, showing that cone outer segments are longer in the transplanted retina than in the control retina.

In the present study, a piece of normal retinal tissue from Sprague Dawley rat (SD) containing either isolated photoreceptors (97% rods) or whole neural retina was transplanted into 3 month-old P23H rat by subretinal insertion. Fundus examination carried out immediately after transplantation revealed retinal detachment induced by the surgery at the site where the graft was placed beneath the neural retina (RD, Figure 1A). One week after transplantation, the retinal detachment had healed as seen in the fundus of the treated eye (Figure 1B) which does not differ from the fellow eye (Figure 1C). Two months after transplantation, fundus fluorescence imaging was performed to visualize the transplant. Only natural autofluorescence can be observed in the fundus of non-transplanted eyes (Figure 1D). However, the transplant (labeled with the fluorescent dye PKH26) was indicated by the hyper autofluorescence, situated in a zone superior to the optic nerve (ON, Figure 1E) in the transplanted eyes. The P23H rats (transplanted at 3 months of age) were sacrificed 6 months later for scoring the survival effect on cones. In transversal sections of the retina (6 months post-transplantation) the presence of the transplant can be detected by anti-rhodopsin antibody staining (green) since the rods of the host animal have degenerated, showing that the transplant is apposed to the inner nuclear layer of the host retina stained in blue with DAPI (Figure 1F). On the flat mounted P23H retina, the grafted tissue was visualized in the upper part of Figure 2A by anti-rhodopsin antibody labeling (red). In both series (whole retina and photoreceptor transplantations) grafts were detected in 100% of the operated eyes, covering less than ¼ of the retinal surface.

**Figure 3:**
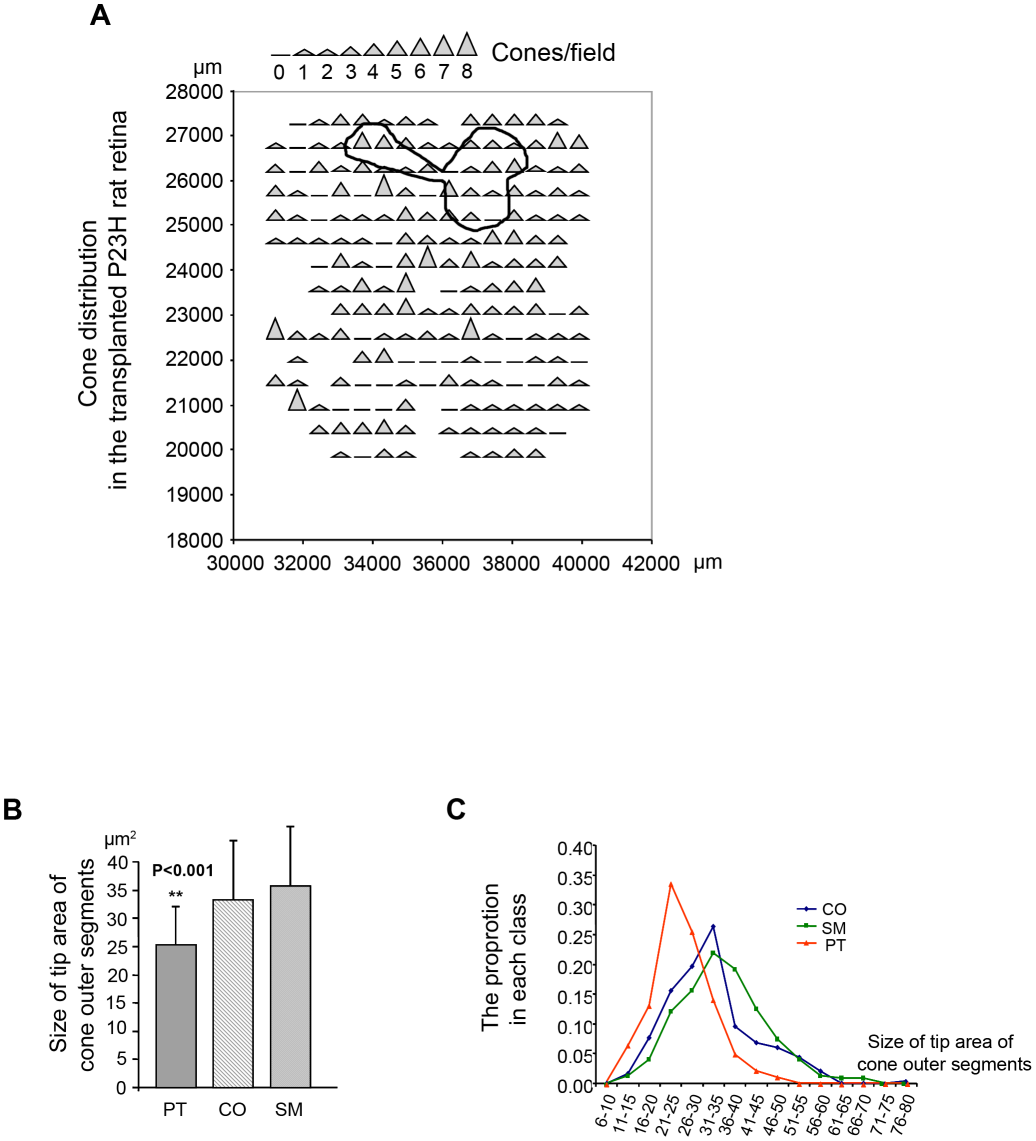
The distribution of cones after retinal transplantation and the size of tip area of cone outer segments. (**A**) This image of cone counting with 193 fields by stereological approach shows the surviving cones distributed in the whole retina, not only localized in the area of transplant. The transplanted area was enclosed by a black line. This image corresponds to Figure 2A. (**B**) Comparison of the size of tip areas of cone outer segments. (**C**) The distribution of the size of tip area of cone outer segments in three experimental groups (photoreceptor transplanted, contralateral control, sham control), corresponding to **B**.

**Figure 4:**
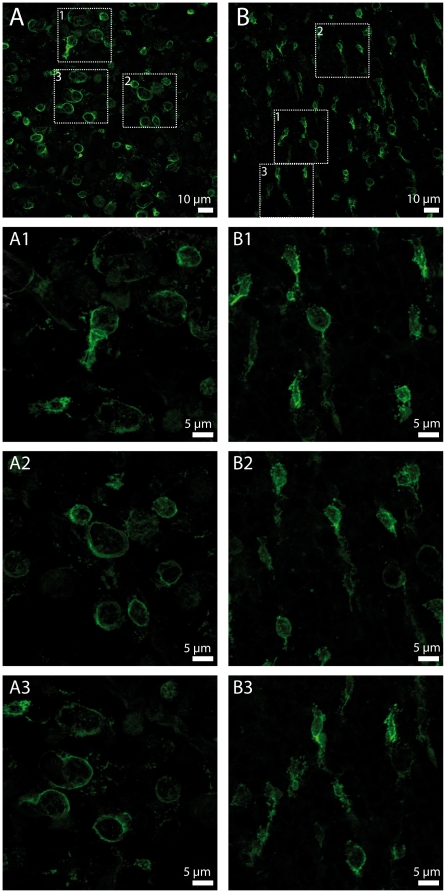
Morphology of cones in the control and photoreceptor transplanted retinas. (A) The cones in the untreated control P23H rat retina present large areas of tip sheath and short outer segments. (B) While, small tip areas associated with long cone outer segments are observed in the photoreceptor transplanted P23H rat retina. A1–A3, B1–B3, images with high magnification corresponding to the small parts in A and B.

We used a stereological counting approach to achieve unbiased sampling, as previously described [29], [30]. The cones were counted and the density of cones (PNA lectin, green) was observed varying regionally over the surface of the grafted retina (see panels 1 and 2, Figure 2A). Image 1 was taken from the nasal-ventral quadrant, while image 2 was taken from the temporal-ventral quadrant, both of them being located at the opposite side of the transplant (located at the dorsal part of the retina). These cones were distributed over the surface of the grafted retina including the non-transplanted areas, but not only localized in the transplanted area, implying that they correspond mainly to the cones from the host retina (Figure 3A). Figure 2B shows two images taken in the same area of the nasal-ventral quadrant, from the control (CO) and photoreceptor transplanted (PT) eye of the same animal. We have noticed that the cone outer segments are longer in the transplanted retinas compared to those of the controls. Moreover, we selected 32 images from each retina in three experimental groups (PT, CO, SM-sham control) at 2 mm away from their optic disc in four directions (dorsal-nasal-ventral-temporal) by stereological acquisition system in order to quantify the morphological variety. On the flat-mounted retinas, the cone morphology of untreated and sham control P23H rat showed enlargement of tip areas (CO, 33.25±10.17 µm^2^; SM, 35.69±10.03 µm^2^) and shortening of cone outer segments, while this phenomenon was reversed by the transplantation of photoreceptors (PT, 25.33±6.98 µm^2^), as demonstrated by the small areas of tip sheath and the long outer segments of cones (Figure 3B, 4 and Table 1). The cone distribution following the different sizes of tip area of cone outer segments suggests that the morphological effect observed in the three experimental groups is not related to the two different types of cones (blue and green) as each curve of the cone distribution contains only one peak, implying that these cones attribute uniquely to one class of cone photoreceptors (Figure 3C). We observed that in the control and sham retinas, the tip areas of cone outer segments were 31% and 41% larger, respectively, than in the photoreceptor transplanted retinas. Using the same method of comparison, we observed when the tip areas of control and sham retinas were found 346% and 378% larger than those of wild type rats (SD, 7.46±1.86 µm^2^, n = 3) at the age of 9 months, they were only 240% larger in photoreceptor transplanted retinas, demonstrating changes (CO/SD – PT/SD  = 146%; SM/SD – PT/SD  = 178%) in cone morphology and a decrease of cone functional damage.

**Table 1:** Size of tip area of cone outer segments in different experimental groups.

Number of retinas	Photoreceptor transplanted retinas (µm^2^)	Control retinas (µm^2^)	Sham retinas (µm^2^)
1	26.54±7.32	32.76±9.46	34.63±10.92
2	23.81±5.92	32.10±9.68	35.34±9.25
3	24.60±5.25	31.64±10.86	37.28±9.12
4	26.64±5.87	33.49±8.12	37.21±9.13
5	30.59±8.13	38.15±12.49	34.11±10.06
Average of five retinas	25.33±6.98	33.25±10.17	35.69±10.03

The data show that the tip areas of cone outer segments in the photoreceptor transplanted retinas are significantly smaller than those in the sham control and their contralateral control retinas (in both P<0.001).

**Figure 5:**
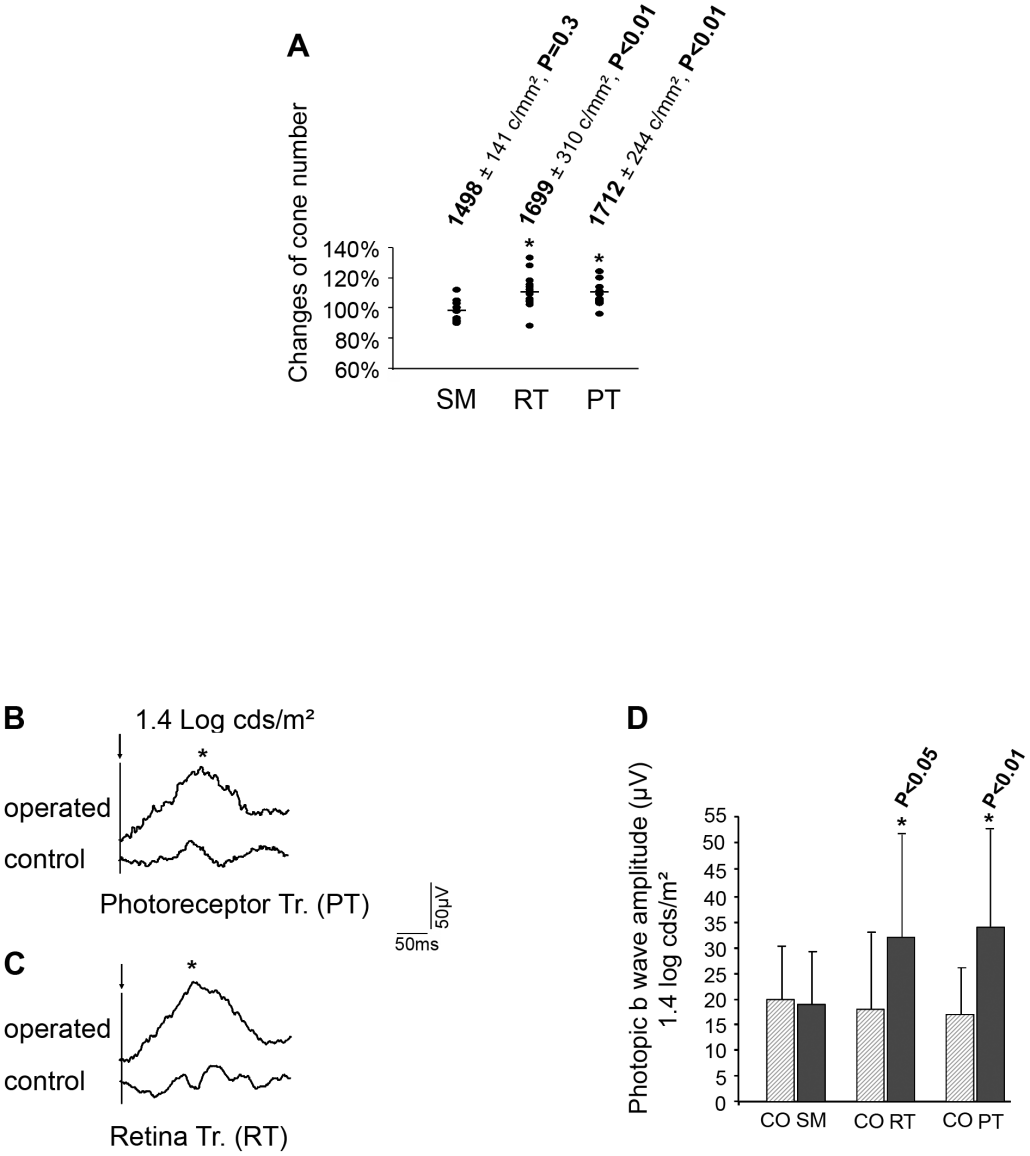
Comparison in the different groups by cell counting and photopic ERG b-wave amplitude. CO: control eye, SM: sham control eye, RT: retinal transplanted eye, PT: photoreceptor transplanted eye. (**A**) Scatter plot shows normalization of changing cones from each rat by comparison of treated eye and its corresponding contralateral control eye. 100% for each control eye. Means of percentage in treated groups, SM: 98%, P = 0.3; RT: 111%, * P<0.01; PT: 111%, * P<0.01. (**B** and **C**) Comparison of photopic ERG average of 5 single flashes from P23H cases after transplantation. b-wave amplitude: **B**, control eye- 33 µV, operated eye- 72 µV. **C**, control eye- 27 µV, operated eye- 86 µV. (**D**) Difference of photopic ERG b-wave amplitudes in each group between the control and treated eyes.

The cone count in the treated retinas was normalized to that obtained from the non-treated contralateral eyes of each animal (Figure 5A). We found no statistically significant difference in the cone density between the sham-operated eyes [2] and their contralateral control eyes (1498±141 c/mm^2^ versus 1535±138 c/mm^2^, n = 10, p = 0.3). By contrast, cone density was statistically significantly higher in the transplanted eyes compared to their contralateral control eyes (total retinal transplantation- RT versus CO, 1699±310 c/mm^2^ versus 1551±175 c/mm^2^, n = 14 p<0.01; PT versus CO, 1712±244 c/mm^2^ versus 1560±196 c/mm^2^, n = 14, p<0.01). The amplitude of this effect (+10%) was similar for both photoreceptors and whole retina transplantation, consistent with the fact that cone rescue is mediated mainly by photoreceptor cells (97% rods) of the transplanted tissue [30].

To evaluate the cone function, we performed ERG recording of the treated animals before sacrifice. It was found that 6 months after surgery, the transplantation using either isolated photoreceptor tissue or total retina promotes a persistence of a photopic b-wave (Figure 5B, C), as demonstrated by an increase of 78% of photopic b-wave amplitudes for retinal transplantation (32±20 µV versus 18±16 µV, p<0.05, n = 14) and of 100% for photoreceptor transplantation (34±18 µV versus 17±9, p<0.01, n = 16; Figure 5D and Supporting Information: Tables S1, S2, S3, S4, S5). Moreover, we compared the ERG photopic b-wave amplitude ratio between these P23H rats and the wild-type rats (SD, 146±24 µV, n = 6) at 9 months and we found that it increased from 12% and 13% in the control and SM groups to 22% (RT) and 23% (PT) in the transplanted groups, demonstrating a rescue of the visual function in transplanted P23H rats in photopic condition.

No effect of the surgery was observed in the sham operated rats (SM versus CO: 19±11 µV versus 20±10 µV). No significant effect from the scotopic responses was found in treated and control rats (b-wave: PT, 29±12 versus 29±9; RT, 28±11, versus 26±7, Table S6, S7).

## Discussion

There is currently no approved treatment for retinitis pigmentosa, except for some syndromic types, in which metabolic disorders can be corrected or minimized by dietary restrictions. So far, a few therapeutic approaches for retinal survival have been proposed and introduced in the clinical practice. However, the evidence for functional rescue is still missing because of feasibility, accessibility, and cost concerns. Recent results from the RPE65 trial offer some promise for gene-specific (or mutation-specific) strategies, with corresponding limitations. Thus, survival of photoreceptors in RP and preservation of their function still remains a major therapeutic challenge.

Among the restorative therapeutic approaches, we present here retinal transplantation as an approach to delay the secondary cone degeneration and, consequently, the central vision loss in RP. The visual handicap induced by RP and other retinal degenerative disorders is effectively related to the loss of photopic functions (central visual acuity, color vision, contrast sensitivity and photopic visual field) supported by the cone photoreceptors, so the preservation of cones by transplantation of normal retinal tissues could be a relevant strategy to combat the blindness related to these affections. Since in the most common forms of RP the causal mutations are located in genes expressed specifically by rods, the secondary degeneration of cones seems likely mediated through non-cell autonomous mechanisms. Using an animal model of recessive RP, the *rd1* mouse, we have previously demonstrated that the rod-cone trophic interactions play a role in the cone degeneration and that the secondary cone degeneration is mediated, at least in part, by the loss of survival signals provided by or requiring the presence of rods [30], [31]. We have also shown that the transplantation of normal rods in the subretinal space of the *rd1* mouse induces a significant host cone survival. Since the trophic approach aims at protecting cells that are not directly affected by the genetic defect, the protection of cones could be applied in the most common forms of RP, such as autosomal dominant RP. The results of the present study validate this concept and demonstrate a benefit of transplantation in transgenic P23H rat. Since the beneficial effect was present 6 months after the transplantation, it can be considered as long-lasting, given the life-time of this species. Furthermore, we have observed no difference of functional effects and cone survival between retinal transplantation and photoreceptor transplantation. Hence, using retinal transplantation will not only facilitate the process of the graft preparation by minimizing tissue manipulations and cell destruction, but also lower the risk of contamination to the transplants, which is more practical in the clinic.

From a clinical point of view, the functional activity of the preserved cells is of crucial importance. In the present study we demonstrate that the protection of cones induced by retinal transplantation is accompanied by some preservation of their function, as demonstrated by the significant persistence of the photopic cone driven ERG recordings. Indeed, the amplitude of the b-wave was increased by 100% and 78% for photoreceptor- and whole retina transplanted eyes, respectively, compared to the corresponding controls. Photopic b-wave amplitude correlated with the survival of cones, but the relationship between both effects was not linear, since the effect on the cone function was more pronounced than the effect on the cone counting. Similar findings were also observed when human embryonic stem cells were transplanted into the subretinal space of adult *Crx* ^−/−^ mice, a model of Leber's congenital amaurosis [32]. The present data may link the effect of transplantation to the morphology of the outer cone segments, as shown by the PNA labeling (Figure 4: small areas of tip sheath and long outer segments). Furthermore, the amplitudes of the photopic ERG b-wave recorded from either selected photoreceptor (34±18 µV) or total retinal (32±20 µV) transplanted eyes indicate that the surviving cones of P23H rats at 9 months can have the same functional activity as those cones at 4 months of age without transplantation (33±9 µV, Supporting Information: Tables S1, S2, S3, S4, S5). The photopic responses reflect exclusively the cone function because these responses have been recorded after rod desensitization (10 minutes light adaptation) in our experiment. As the rod photoreceptors represent the majority of the photoreceptors of the transplant (97%), and the size of the transplant does not exceed 20% of the host retina surface, it is unlikely that a few donor cones could be enough to drive such an improvement in the photopic b-wave recorded, even though these cells might form some synaptic connections with the host inner nuclear layer cells. Since ERG measurement depends on the capture of the photon by opsin molecules located in outer segments, the preservation of this structure, in addition to the cone survival, the changes in bipolar or horizontal cell function to photoreceptor input, and synaptic connectivity from the surviving cones, is likely to underlie the synergy scored by the ERG. One could even suggest that the survival of cones is a result of the maintenance of their function, since active neurons are more likely to survive. In addition, we have not observed significant changes of ERG scotopic a-wave and b-wave. These effects also reflect the molecular mechanism involved only in the protection of cones. The cone distribution following the sizes of tip area of cone outer segments in the three experimental groups suggests that the majority of the cones belongs to one class of cone photoreceptors, because each curve of distribution contains only one peak. Szel and Rohlich [33] have observed that in rat retinas, green cones constitute the large majority (about 93%) of cones, while blue cones make up only 7%. Therefore, the majority of the cones counted in our experiments are green cones and we think that the morphological difference between blue and green cones seems unable to account for the differences observed in our experimental groups.

We demonstrate here that the survival of cones does not depend on direct contact between the grafted tissue and the surviving host cones, since the cones are distributed over the entire surface of the retina. A gradient of survival effect from the transplant to the other zones away from it has not been clearly observed (Figure 3A). Differences of cone number in the four quadrants of some retinas have occasionally been found, but it is difficult to state that this phenomenon represents a gradient of survival effect or a gradient of degeneration, since the graft position was not taken into consideration during the cell counts in all the samples. Further experiments are needed to specifically address these questions. Our analysis suggests that the entire effect is mediated by molecules originating from the photoreceptor cells of the transplanted tissue (Figure 5A). Although the results presented here do not address directly the nature of these trophic molecules, rod-derived cone viability factors RdCVF and RdCVF2 [34], [35] seem to be possible candidates. Synthetic RdCVF protein has been shown to protect, both morphologically and functionally, the P23H rat cones with maintenance of the outer cone segments [2]. The RdCVF-induced effect on cone function was more pronounced than those on cone survival, in proportion similar to the one observed in the present study. The products of the *Nxnl1* gene, encoding RdCVF, could be involved in the pathophysiological mechanisms leading to secondary cone degeneration in RP. This suggestion is in accordance with the fact that the *Nxnl1*−/− mouse displays a progressive loss of cones [36]. The molecular mechanism linking the secreted truncated nucleoredoxin-like protein RdCVF to the effect on the morphology of the outer segment remains unknown. It could involve an indirect mechanism related to the metabolism of cone cells that are starving after rod death [37] or, more directly, a mechanism requiring the interaction of the *Nxnl1* gene product with the microtubule interacting protein TAU [38], or both. Further investigations are needed to answer this question.

In conclusion, the present study provides evidence for efficient and long-term functional rescue of the cone function in P23H rat model of autosomal dominant RP. It seems feasible that the results presented here could be transferable to the clinic, presuming that the loss of 50% cones does not result in significant loss of visual acuity [39]. In addition, it has been stated that keeping the functional cones alive, may potentially prevent 1.5 million people worldwide from becoming blind [40]. The question remains whether transplantation of retinal tissues or the delivery of trophic molecules will be the most practicable.

## Materials and Methods

### Animals

All procedures were carried out according to the Statement of the Association for Research in Vision and Ophthalmology Statement for the Use of Animals in Ophthalmology and Vision Research. Transgenic homozygous P23H rats (line 1) [41] were kindly provided by Matthew LaVail (UCSF School of Medicine, Beckman Vision Center, San Francisco, CA) and were crossed with albino Sprague-Dawley rats purchased from Charles-River (Saint Aubin Les Elbeuf, France) to produce heterozygous animals. All animals were kept under a 12-hour light/dark cycle with light intensity of 15 lux and room temperature at 25°C. They were housed with the authorization and supervision of the institutional Animal Care from Inserm U968. The animals were divided into 3 groups and the treated eyes were randomly selected. Group 1, n = 10: 3-month-old rats for sham operation; Group 2, n = 14: 3-month-old rats for photoreceptor transplantation; Group 3, n = 14: 3-month-old rats for entire retinal transplantation. The treated eyes were randomly selected. The contralateral unoperated eye from each rat was considered as its natural control. The animals were sacrificed by anesthesia overdose using 2–2.5 ml ketamine 500 (Imalgène Rhone Merieux, Lyon, France). All experiments were performed with the approval of the Ethics Committee for Animal Experimentation Charles Darwin (N°: Ce5/2009/048).

### Transplantation

#### Photoreceptor transplantation

Sheets of photoreceptors (97% rods) were prepared from postnatal 8 day Sprague-Dawley rats by vibratome sectioning, as previously described [30], [42]. The remaining sheet of photoreceptor stuck on a piece of gelatin (total 250–300 µm thickness) was kept in culture medium (CO_2_ independent medium without L-glutamine, Sigma) at 4°C overnight before grafting. During the operation, small pieces of this tissue (3 mm×2 mm) were cut and prepared for transplantation. To label photoreceptors by the fluorescent dye PKH26 (green fluorescent general cell linker kit, Sigma, St. Louis, MO), photoreceptor sheets were placed in the culture medium in which PKH26 (4 µl/ml) was dissolved for 5 to 10 minutes according to manufacturer instructions.

#### Retinal transplantation

Rats were anesthetized by intramuscular injection of a mixture of ketamine (100 mg/kg) and xylazine (10 mg/kg). For transplantation of the entire retina (both outer and inner layers), postnatal 8 day-old neural retinas were dissected and small pieces were grafted. A small scleral hole was made after conjunctival incision and a local retinal detachment was induced by phosphate buffer saline injection. Subsequently, the scleral incision was enlarged. The subretinal injector was carefully inserted and the grafted tissue was released. The scleral hole was then closed with a 10-0 needle suture.

All the transplants were inserted in the dorsal quadrants of the host retinas. Fundus photos and autofluorescence images of the transplanted P23H eyes and their contralateral control eyes were obtained respectively at 1 week and at 2 months after transplantation by using a Heildelberg Retina Angiograph 2.

### Electroretinography (ERG) recordings

The ERG recording was performed according to Yang et al. [2]. Briefly, following 12 hours dark adaptation under dim red light, animals were anesthetized by intramuscular administration of a mixture of ketamine (100 mg/kg) and xylazine (10 mg/kg). Pupils were dilated with 0.5% tropicamide or 1% atropine. Body temperature was maintained at approximately 37°C with a heating pad. ERGs were recorded from both eyes by using a gold loop electrode on the corneal surface and maintained with lubricating ointment Lacrigel (Europhta, Monaco). Two stainless steel reference electrodes were inserted subcutaneously on the two sides of the head. A third needle electrode inserted subcutaneously in the back of the rat served to ground the signal. The light stimulus, provided by a 150 Watt xenon lamp in a Ganzfeld stimulator (Multiliner Vision, Jaeger Toennies, Germany) was increased from −4 to 1.4 log cd/m^2^. Photopic cone ERGs were performed on a rod suppressing background after 10 minute light adaptation. Each light stimulus was applied for 20 seconds. Responses were amplified and filtered (1 Hz-low and 300 Hz-high cut off filters) with a 1-channel DC-/AC amplifier. The amplitude and latency of a-wave and b-wave were measured from the average of five responses by a set of five flashes of stimulation.

### Immunohistochemistry

After enucleation, the eyes were immersed at 4°C overnight in 4% paraformaldehyde in PBS 0.01 M, pH 7.4. The tissues were successively incubated for 2 hours in 10% and 20% sucrose at 4°C for cryoprotection, and embedded in optimum cutting temperature medium to prepare for cryostat sectioning. In order to obtain flat mounted retinas, the eyes were enucleated and quickly immersed into PBS. The anterior segment, lens, and vitreous body were removed. The neural retinas were initially fixed in 4% paraformaldehyde for 2 hours at 4°C and rinsed in PBS 3 times (5 minutes each). The sectioned retina tissues after PBS washing or the total neural retinas were permeabilized for 5 minutes in PBS containing 0.1% Triton X-100, and incubated in PBS containing 1% Bovine serum albumin and 0.1% Tween-20 for 30 minutes at room temperature. Anti-rhodopsin antibody (dilution 1∶250) and PNA lectin from Arachis hypogae (FITC-conjugated peanut agglutinin, dilution 1∶50) were incubated overnight at 4°C to label rod and cone photoreceptor cells respectively. After washing, sectioned tissues or total neural retinas were incubated with a secondary antibody: goat anti-mouse IgG conjugated to Alexa TM 594 or 488 labeled antibodies at 1∶500 dilutions for 1 hour. The nuclear marker 4′-6-diamidino-2-phenylindole- DAPI was added to the incubation solution only for sectioned tissues which were ultimately mounted with Flurosave reagent (Calbiochem, San Diego, CA). Photoreceptors of the total neural retinas were kept facing up on the slide and the retinas were flat mounted in gel medium for microscopic analysis (Biomeda, Foster City, CA).

### Cell counts and measurement of the tip areas of cone outer segments

In order to achieve unbiased sampling, the total number of PNA-labeled cones was estimated in the flat-mounted retinas by using the stereological counting approach previously described [29], [30]. The cells were counted on nearly 200 sampled non overlapping 1225 µm^2^ zones determined in a systematic random fashion to sample equally the whole retinal surface (extending from the center of the optic nerve head over a radius of 4 mm). The number of cones was visually counted by using a Nikon Plan 40× objective on a Nikon photomicroscope equipped with a Sony Trinitron color graphic display camera (Sony, Tokyo).

We chose five retinas of each group from control, sham and photoreceptor transplanted retinas in order to compare the tip areas of cone outer segment. 32 images from each retina were selected at 2 mm away from their optic disc in four directions (dorsal-nasal-ventral-temporal) by stereological acquisition system. The size of the tip areas of cone outer segments which sited in the central zone (1225 µm^2^) of each image was measured in a masked analysis. The comparison of the tip areas in these retinas was shown in Figure 3B and Table 1.

### Statistical analysis

The method of Kolmogorov-Smirnov test was performed to evaluate whether the data followed a normal distribution. Student's t test was performed for paired series of ERG and cell counts. All the samples for ERG and cell counts were paired to compare treated versus control retinas in each group (cone number of each mm^2^ per retina, amplitude and latency of ERG a- and b- waves separately). Data from ERG, cone numbers and tip area of cone outer segments are presented as mean (X) ± standard deviation (S). Differences were considered to be significant at P<0.05.

## Supporting Information

Table S1 Photopic b-wave amplitude and latency, and cone count of the photoreceptor transplanted and contralateral control P23H rat eyes.
(0.06 MB DOC)

Table S2 Photopic b-wave amplitude and latency, and cone count of the retina transplanted and contralateral control P23H rat eyes.
(0.06 MB DOC)

Table S3 Photopic b-wave amplitude and latency, and cone count of the sham operated P23H rat eyes.
(0.04 MB DOC)

Table S4 Averages of photopic b-wave amplitude and latency, and cone count in the treated and corresponding control eyes.
(0.04 MB DOC)

Table S5 Photopic ERG b-wave amplitude of P23H rats at 4 months without transplantation.
(0.04 MB DOC)

Table S6 Scotopic ERG b-wave amplitude and latency of the photoreceptor transplanted and contralateral control P23H rat eyes.
(0.04 MB DOC)

Table S7 Scotopic ERG b-wave amplitude and latency of the retina transplanted and contralateral control P23H rat eyes.
(0.04 MB DOC)
