# Supplementary material for: Transplantation of Photoreceptor and Total Neural Retina Preserves Cone Function in P23H Rhodopsin Transgenic Rat
Source: PLoS One. 2010 Oct 19;5(10):e13469. doi: 10.1371/journal.pone.0013469 (PMC2957406; doi:10.1371/journal.pone.0013469)
Supplement: Table S1 — Photopic b-wave amplitude and latency, and cone count of the photoreceptor transplanted and contralateral control P23H rat eyes. (0.06 MB DOC) [file pone.0013469.s001.doc]

**Supplemental table 1**: Photoreceptor transplantation (operated at 3 month age, sacrificed at 9 month age)

| Number of rats | Photopic ERG b-wave amplitude (µV)  **operated eye** | Photopic ERG b-wave amplitude (µV)  **control eye** | Photopic ERG b-wave latency  (ms)  **operated eye** | Photopic ERG b-wave latency  (ms)  **control eye** | Cone counts  (cells /mm^2^)  **operated eye** | Cone counts  (cells /mm^2^)  **control eye** |
| --- | --- | --- | --- | --- | --- | --- |
| 1 | 45.5 | 20.6 | 99.6 | 144.0 | 1730 | 1649 |
| 2 | 60.0 | 22.7 | 92.4 | 93.5 | 1967 | 1641 |
| 3 | 37.5 | 3.8 | 74.4 | 97.2 | 1527 | 1404 |
| 4 | 42.1 | 20.5 | 92.4 | 114.0 | 1845 | 1486 |
| 5 | 71.7 | 32.7 | 114.0 | 92.4 | NA | NA |
| 6 | 27.3 | 36.4 | 100.8 | 104.4 | 1820 | 1592 |
| 7 | 22.7 | 15.9 | 135.6 | 56.4 | 1722 | 1796 |
| 8 | 45.5 | 2.0 | 106.8 | 128.4 | 2041 | 1845 |
| 9 | 45.2 | 20.9 | 93.6 | 81.6 | NA | NA |
| 10 | 18.6 | 18.3 | 94.8 | 106.8 | 2057 | 1665 |
| 11 | 18.3 | 22.0 | 112.8 | 104.4 | 1812 | 1747 |
| 12 | 14.5 | 17.4 | 94.8 | 106.8 | 1469 | 1380 |
| 13 | 11.4 | 3.7 | 111.6 | 109.2 | 1249 | 1208 |
| 14 | 47.5 | 11.8 | 104.4 | 110.4 | 1616 | 1714 |
| 15 | 20.2 | 12.5 | 108.4 | 103.2 | 1747 | 1404 |
| 16 | 13.8 | 16.2 | 80.4 | 99.6 | 1363 | 1306 |
