# Supplementary material for: Transplantation of Photoreceptor and Total Neural Retina Preserves Cone Function in P23H Rhodopsin Transgenic Rat
Source: PLoS One. 2010 Oct 19;5(10):e13469. doi: 10.1371/journal.pone.0013469 (PMC2957406; doi:10.1371/journal.pone.0013469)
Supplement: Table S2 — Photopic b-wave amplitude and latency, and cone count of the retina transplanted and contralateral control P23H rat eyes. (0.06 MB DOC) [file pone.0013469.s002.doc]

**Supplemental table 2**: Retinal transplantation (operated at 3 month age, sacrificed at 9 month age)

| Number of rats | Photopic ERG b-wave amplitude (µV)  **operated eye** | Photopic ERG b-wave amplitude (µV)  **control eye** | Photopic ERG b-wave latency  (ms)  **operated eye** | Photopic ERG b-wave latency  (ms)  **control eye** | Cone counts  (cells /mm^2^)  **operated eye** | Cone counts  (cells /mm^2^)  **control eye** |
| --- | --- | --- | --- | --- | --- | --- |
| 1 | 58.7 | 8.0 | 120.0 | 157.2 | 2138 | 1807 |
| 2 | 42.0 | 1.9 | 130.8 | 97.2 | 1951 | 1528 |
| 3 | 32.9 | 3.4 | 129.6 | 91.2 | 1878 | 1670 |
| 4 | 41.0 | 43.2 | 125.2 | 118.1 | 1510 | 1486 |
| 5 | 86.0 | 26.7 | 98.4 | 139.2 | 2261 | 1706 |
| 6 | 26.1 | 36.9 | 66.0 | 122.4 | 1396 | 1589 |
| 7 | 20.7 | 30.1 | 98.4 | 146.6 | 1371 | 1257 |
| 8 | 27.0 | 11.2 | 129.6 | 148.8 | 1633 | 1725 |
| 9 | 15.4 | 0.1 | 99.6 | 96.0 | 1788 | 1632 |
| 10 | 18.2 | 2.7 | 148.8 | 94.8 | 1290 | 1238 |
| 11 | 23.1 | 3.7 | 127.2 | 90.0 | 1640 | 1556 |
| 12 | 19.0 | 21.4 | 88.8 | 99.6 | 1698 | 1496 |
| 13 | 15.0 | 10.1 | 159.6 | 145.2 | 1942 | 1682 |
| 14 | 28.4 | 45.6 | 157.2 | 116.4 | 1290 | 1340 |
