# Supplementary material for: Transplantation of Photoreceptor and Total Neural Retina Preserves Cone Function in P23H Rhodopsin Transgenic Rat
Source: PLoS One. 2010 Oct 19;5(10):e13469. doi: 10.1371/journal.pone.0013469 (PMC2957406; doi:10.1371/journal.pone.0013469)
Supplement: Table S3 — Photopic b-wave amplitude and latency, and cone count of the sham operated P23H rat eyes. (0.04 MB DOC) [file pone.0013469.s003.doc]

**Supplemental table 3**: Sham operation (operated at 3 month age, sacrificed at 9 month age)

| Number of rats | Photopic ERG b-wave amplitude (µV)  **operated eye** | Photopic ERG b-wave latency (ms)  **operated eye** | Cone counts (cells /mm^2^)  **operated eye** |
| --- | --- | --- | --- |
| 1 | 7.5 | 128.4 | 1320 |
| 2 | 13.0 | 108.0 | 1380 |
| 3 | 26.2 | 97.2 | 1550 |
| 4 | 22.4 | 122.4 | 1507 |
| 5 | 37.1 | 94.8 | 1678 |
| 6 | 22.7 | 99.6 | 1400 |
| 7 | 20.9 | 104.1 | 1367 |
| 8 | 8.5 | 93.6 | 1649 |
| 9 | 27.3 | 91.2 | 1707 |
| 10 | 2.5 | 98.4 | 1423 |

ERG photopic b- wave amplitude and latency at intensity 25 cds/m². Cell counts showing cone number per mm² in the retina by stereology approach.
