# Supplementary material for: Transplantation of Photoreceptor and Total Neural Retina Preserves Cone Function in P23H Rhodopsin Transgenic Rat
Source: PLoS One. 2010 Oct 19;5(10):e13469. doi: 10.1371/journal.pone.0013469 (PMC2957406; doi:10.1371/journal.pone.0013469)
Supplement: Table S4 — Averages of photopic b-wave amplitude and latency, and cone count in the treated and corresponding control eyes. (0.04 MB DOC) [file pone.0013469.s004.doc]

**Supplemental table 4**: Photopic ERG and cone counts in different groups

| Groups of rats | Photopic ERG b-wave amplitude (µV)  X ± S | Photopic ERG b-wave latency (ms)  X ± S | Cone counts  (cells/mm2)  X ± S |
| --- | --- | --- | --- |
| Photoreceptor transplantation group: **operated** | 34 ± 18 | 101 ± 14 | 1712 ± 244 |
| Photoreceptor transplantation group: **control** | 17 ± 9 | 103 ± 19 | 1560 ± 196 |
| Retinal transplantation group: **operated** | 32 ±20 | 120 ± 27 | 1699 ± 310 |
| Retinal transplantation group:  **control** | 18 ±16 | 119 ± 24 | 1551 ± 175 |
| Sham:  **operated** | 19 ± 11 | 104 ± 12 | 1498 ± 141 |
| Sham:  **control** | 20 ± 10 | 103 ± 16 | 1535 ± 138 |
