# Supplementary material for: Transplantation of Photoreceptor and Total Neural Retina Preserves Cone Function in P23H Rhodopsin Transgenic Rat
Source: PLoS One. 2010 Oct 19;5(10):e13469. doi: 10.1371/journal.pone.0013469 (PMC2957406; doi:10.1371/journal.pone.0013469)
Supplement: Table S5 — Photopic ERG b-wave amplitude of P23H rats at 4 months without transplantation. (0.04 MB DOC) [file pone.0013469.s005.doc]

**Supplemental table 5**: Photopic ERG b-wave of P23H rats

at 4 months without transplantation

| Number of rats | Photopic ERG b-wave amplitude (µV) |
| --- | --- |
| 1 | 24.1 |
| 2 | 36 |
| 3 | 28.9 |
| 4 | 54.6 |
| 5 | 33 |
| 6 | 40.3 |
| 7 | 28.5 |
| 8 | 36.2 |
| 9 | 27.6 |
| 10 | 25.7 |

X ± S = 33 ± 9 µV
