# Supplementary material for: Transplantation of Photoreceptor and Total Neural Retina Preserves Cone Function in P23H Rhodopsin Transgenic Rat
Source: PLoS One. 2010 Oct 19;5(10):e13469. doi: 10.1371/journal.pone.0013469 (PMC2957406; doi:10.1371/journal.pone.0013469)
Supplement: Table S6 — Scotopic ERG b-wave amplitude and latency of the photoreceptor transplanted and contralateral control P23H rat eyes. (0.04 MB DOC) [file pone.0013469.s006.doc]

| Number of rats  **Supplemental table 6**: Photoreceptor transplantation (operated at 3 month age, sacrificed at 9 month age) | Scotopic ERG  b-wave  amplitude (µV)  **operated eye** | Scotopic ERG  b-wave  amplitude (µV)  **control eye** | Scotopic ERG  b-wave  latency (µV)  **operated eye** | Scotopic ERG  b-wave  latency (µV)  **control eye** |
| --- | --- | --- | --- | --- |
| 1 | 16.6 | 15.4 | 114 | 116.6 |
| 2 | 44.3 | 32.5 | 118.8 | 106 |
| 3 | 13.4 | 25.4 | 108.6 | 102 |
| 4 | 27.2 | 30.5 | 122 | 112.4 |
| 5 | 52.7 | 41.9 | 111.6 | 114 |
| 6 | 15.1 | 20.6 | 116 | 98.4 |
| 7 | 16.2 | 41.9 | 96 | 107.4 |
| 8 | 31.7 | 14.4 | 124.8 | 118 |
| 9 | 45.2 | 39.8 | 120 | 94 |
| 10 | 26.8 | 21.7 | 115.2 | 103.6 |
| 11 | 19.1 | 27.7 | 112.2 | 118.8 |
| 12 | 32.5 | 25.7 | 121 | 116 |
| 13 | 25.5 | 30.6 | 115.8 | 112.4 |
| 14 | 45.7 | 38.3 | 104.4 | 122 |
| 15 | 28.4 | 19.8 | 115.2 | 107.4 |
| 16 | 22.8 | 32.3 | 98 | 101.4 |
