# Supplementary material for: Transplantation of Photoreceptor and Total Neural Retina Preserves Cone Function in P23H Rhodopsin Transgenic Rat
Source: PLoS One. 2010 Oct 19;5(10):e13469. doi: 10.1371/journal.pone.0013469 (PMC2957406; doi:10.1371/journal.pone.0013469)
Supplement: Table S7 — Scotopic ERG b-wave amplitude and latency of the retina transplanted and contralateral control P23H rat eyes. (0.04 MB DOC) [file pone.0013469.s007.doc]

| Number of rats  **Supplemental table 7**: Retinal transplantation (operated at 3 month age, sacrificed at 9 month age) | Scotopic ERG  b-wave  amplitude (µV)  operated eye | Scotopic ERG  b-wave  amplitude (µV)  control eye | Scotopic ERG  b-wave  latency (µV)  operated eye | Scotopic ERG  b-wave  latency (µV)  control eye |
| --- | --- | --- | --- | --- |
| 1 | 45.5 | 27.8 | 109.2 | 106.6 |
| 2 | 23.7 | 32.4 | 106.8 | 114 |
| 3 | 36.7 | 13.4 | 116.6 | 121.2 |
| 4 | 29.2 | 28.8 | 113.5 | 122 |
| 5 | 52.2 | 32.7 | 112 | 110.4 |
| 6 | 19.4 | 21.6 | 112.8 | 118.4 |
| 7 | 19.6 | 34.6 | 97.2 | 102 |
| 8 | 25.1 | 20.9 | 99.2 | 112 |
| 9 | 17.9 | 25.8 | 104 | 100.8 |
| 10 | 22.7 | 32.5 | 112 | 118 |
| 11 | 31.7 | 24.8 | 103.4 | 106 |
| 12 | 29.6 | 13.8 | 98 | 106 |
| 13 | 13.8 | 26.4 | 113.5 | 97.6 |
| 14 | 28.4 | 35.1 | 98 | 112 |
